# The Hospital World

**Published:** 1910-07-23

**Authors:** 


### The Hospital World.

### Elections and Appointments.

Sir Edward Wood has been re-elected chairman, and Sir Arthur Hazlerigg, Bart., vice-chairman (in place of Mr. G. Murray Smith), of the Leicester Infirmary at the recent half-yearly meeting of Governors.

Mr. G. Kinton has been elected Chairman of the Hinckley Joint Hospital Board for the ensuing year. The Visiting Committee, as enlarged, now consists of Colonel Harris, Mr. G. Kinton, Mr. G. Geary, Mr. C. Hands, and Mr. W. Johnson.

Mrs. George Sneath has been elected a Life Governor of Leicester Infirmary in recognition of Mr. George Sneath's donation of £100, and Mr. Henry Clarke also has been elected a Life Governor in respect of the public commemoration-gift of £100 from the Leicester Co-operative Society, Ltd.

Mr. W. H. Dakin, Mr. Sydney Cozens-Hardy, and Dr. Arthur Greene, members of the Committee of Management of the Norfolk and Norwich Eye Infirmary, have been co-opted members of the Extension Committee for dealing with the erection of the new Eye Infirmary at the Norfolk and Norwich Hospital.

Mr. T. T. L. Overend, having contributed £21, has been elected a Life Governor of the Royal Hospital for Incurables, Dublin, and Mr. John Halligan has been co-opted a member of the Committee of Management to fill the vacancy caused by the death of the late Mr. Thomas Pigot. The visiting governors for the month have been appointed as follows: Mrs. O'Connor, Miss Mooney, Alderman George Healy, J.P., and Mr. Laurence Mulligan, J.P.

Mr. Hirst, Mr. Bradshaw, the Rev. R. J. Foster, Mr. Hunt, and Mr. Clowes have been elected to form the House Committee for the ensuing year at the Retford Hospital and Dispensary, Notts. The Rev. T. Morgan, the Rev. L. V. Rowarth, the Rev. J. T. Bumford, and Mr. Scott have been appointed a sub-committee to check the dispensary patients' book in order to prevent the use of the dispensary by ineligible cases. It is hoped that this Committee may lead either to the appointment of an almoner, or may itself fulfil the object of that office.

The Board of Management of the Royal Albert Edward Infirmary, Wigan, has been chosen as follows: Mr. D. Goldsworth (the treasurer), with the President, Life Vice-Presidents, Vice-Presidents, and Mr. John Dean, Mr. Joseph Parkinson, Mr. John Knowles, Mr. R. O. Burland, Mr. O. P. Lancashire, Mr. Wm. Johnson, Mr. John Wood, Mr. James Wilson, Mr. E. Walkden, Mr. Joseph T. Gee, Mr. J. M. Lister, Mr. William Rigby, Mr. Thorley Smith, Mr. Ernest Douglas, Mr. J. Peterkin, Mr. Wm. Black, Mr. H. V. Athron, Mr. Robert Layland, and four of the hon. medical officers.

Mr. Henry Joslin, J.P., D.L., of Upminster, has been appointed president of the Romford Victoria Cottage Hospital, Essex, Mr. Ernest Winmill has been re-elected hon. treasurer, and Mr. W. K. James and Mr. B. Keeble hon. secretaries. Mr. Keeble, however, is shortly to resign, and has only accepted re-election for the sake of the hospital till new arrangements have been made. In the meantime Mr. F. Chipperfield, Mr. J. T. Stratford, Mr. A. C. Powell, Mr. W. J. Harvey, Mr. Ernest Winmill, Mr. B. Keeble, Mr. J. W. Lasham, Mr. N. T. N. King, and Mr. E. J. Colyer are acting as a sub-committee.

The Vicar of Yarmouth, the Rev. Canon J. W. Willink, has been re-elected President, Mr. Alderman W. Barnard, Vice-President, and Mr. Councillor R. F. E. Ferrier, Hon. Secretary of the Yarmouth General Hospital. Members of the Committee have been elected as follows: Mr. C. N. Brown, Mr. J. S. Shearman, and Mr. R. G. Westmacott, for the town, and General Upcher for the country.

### Personalia.

The death is announced of Mr. George Wallis, L.D.S., R.C.S., who had been in practice for over fifty years. Mr. George Wallis was one of the founders of the Central London Throat and Ear Hospital, and took the greatest interest in the welfare of the institution. He was a member of the committee from the foundation, and during the past four years was the chairman. As the Central London was founded in 1874, it will be seen that Mr. Wallis devoted the greater part of his life to his hospital, and it is by no means only the friends his practice brought to him who are now feeling his death. As chairman of the hospital round which his interests had centred, Mr. Wallis was able to give authoritative expression to his affectionate interest in its welfare, and probably there is no hospital in London in which this personal relation of chairman to hospital was more intimate.

We learn from Leicester Infirmary of the retirement of Mr. C. J. Bond, F.R.C.S., the Senior Surgeon, from the consulting practice which his great reputation has built up. The Governors of the Infirmary "view with regret and even concern" this news, much as they appreciate Mr. Bond's natural wish for a quieter life after the long period of activity he has spent in the service of others. He will still place at the disposal of his old hospital his experience and talents, however, which have done so much to maintain its reputation. Another worker at the hospital, Miss Gertrude Rogers, the Lady Superintendent of twenty-six years' standing, the Governors hope to reward by stating how much they would welcome the announcement that the Board had decided to associate the devoted service of Miss Rogers with the great reconstruction scheme just carried out. The Governors would approve most cordially the christening of the top ward of the new wing the "Gertrude Rogers ward."

We have recorded more than once in this column the family associations with which hospitals are often connected, particularly in the office of president. It is pleasant now to add an example of connection between a family and the office of secretary. Ever since 1845 the appointment of secretary to the Lincoln County Hospital has been in the Danby family. The hospital is losing the services of Mr. W. B. Danby, who since 1881 has been its secretary, and who succeeded his father after the latter had held it for thirty-six years. In recognition of his constant and loyal service the governors have taken the opportunity afforded by a vacancy in the list of vice-presidents and have elected Mr. Danby to fill the place of the late Marquis of Ripon, so that he will still have a place upon the weekly board. Mr. R. H. Lynn, who as clerk has been a fellow worker with Mr. Danby, received at the recent meeting a deserved tribute to his work, which has been none the less valued because of the detail it involves. Mr. W. Watkins, who for no fewer than thirty-two years has been surveyor to the hospital, is also severing his connection with the institution, to which he has given a very large share of his time. The weekly board has not forgotten to record his work and its appreciation. It speaks well for an institution to keep its workers so long, and they and it have established a tradition to be proud of.

